# Identifying Risk Factors for Low Piglet Birth Weight, High Within-Litter Variation and Occurrence of Intrauterine Growth-Restricted Piglets in Hyperprolific Sows

**DOI:** 10.3390/ani11092731

**Published:** 2021-09-18

**Authors:** Kristina V. Riddersholm, Ida Bahnsen, Thomas S. Bruun, Leonardo V. de Knegt, Charlotte Amdi

**Affiliations:** 1 Department of Veterinary and Animal Sciences, Faculty of Health and Medical Sciences, University of Copenhagen, 1870 Frederiksberg, Denmark; riddersholm95@hotmail.com (K.V.R.); idabahnsen@yahoo.dk (I.B.); lvdk@sund.ku.dk (L.V.d.K.); 2 SEGES Danish Pig Research Centre, 1609 Copenhagen, Denmark; thsb@seges.dk

**Keywords:** feeding system, gestation, hyperprolific sows, IUGR piglets, piglet birth weight, risk factors, within-litter variation in piglet birth weight

## Abstract

**Simple Summary:**

Piglet mortality is an ongoing concern for pig production worldwide. Piglets that have a low piglet birth weight (PBW), suffer from intrauterine growth restriction (IUGR) or are born from litters with a high within-litter variation in PBW (CV_PBW_) have an increased risk of dying before weaning. IUGR piglets, CV_PBW_ and a low PBW might be connected by the same risk factors, and in order to optimize fetal development in the litter, these risk factors should be identified. Free-access stall feeding, floor feeding and electronic sow feeding systems are commonly used feeding systems for gestating sows in Denmark. These systems differ in several points, including in sow competition at feeding. The nutritional status of the sow is important for fetal development, and so the feeding method during gestation is also expected to affect such development. Of the risk factors identified in this study, increasing litter size was considered the most critical. Only small differences were found between the feeding systems and these differed amongst groups. The results should inspire further investigation of those risk factors to clarify causes of the observed effects and what drives individual herd differences.

**Abstract:**

This study aimed to identify risk factors affecting PBW, high CV_PBW_ and the occurrence of IUGR piglets in 12 commercial Danish herds with hyperprolific sows using free-access stalls, floor or electronic sow feeding systems in the gestation unit. The following factors were investigated: the duration of previous lactation, the length of the interval from weaning to insemination, the length of gestation, litter size, parity, sow backfat thickness in late gestation and the type of feeding system in the gestation unit. The study included newborn piglets from 452 litters with the following production indicator averages: 21.3 piglets/L, 1235 g PBW, 22.9% CV_PBW_ and 10.9% and 11.8% within-litter occurrence of severe and mild IUGR piglets, respectively. Increasing length of weaning-to-insemination interval decreased PBW by 25.8 g/day. For 2nd to 9th parity sows, each additional piglet in the litter increased CV_PBW_ by 0.38%, the occurrence of severe IUGR piglets by 0.68% and mild IUGR piglets by 0.50%. Sows of 5th parity and older had a 1.39% higher CV_PBW_ and 49.1 g lighter piglets compared with sows of 2nd to 4th parity. PBW was lower in one ESF herd, suggesting complex interactions that need to be further elucidated. The main critical risk factor observed was litter size.

## 1. Introduction

Over the years, Danish pig production has become more efficient, with a remarkable increase in litter size from 11.8 total born piglets/litter in 1992 to 19.6 total born piglets/litter in 2020 [1]. However, piglet mortality before weaning has not declined accordingly [1], even though the selection criterion was changed from litter size (total number born) to LP5 (number of live piglets at day 5) in 2004 and remains a concern of the industry. Around 20–32% of all piglets have suffered from intrauterine growth restriction (IUGR) during fetal development [2,3], and both IUGR piglets [3,4] and piglets with a low piglet birth weight (PBW) [4,5] have an increased risk of dying [3,4,5]. A high within-litter variation in birth weight (CV_PBW_) is also negatively correlated with survival until weaning [6], which suggests that high piglet mortality is due to litters characterized by a low PBW, high CV_PBW_ and the occurrence of IUGR piglets.

PBW, CV_PBW_ and IUGR piglets might be connected by the same risk factors, and in order to optimize fetal development in litters, those risk factors should be identified. Previous studies have shown that CV_PBW_ is negatively affected by a weaning-to-insemination interval (WII) of less than eight days, when compared to above 21 days [7], while litter size is affected by the length of the previous lactation [8,9,10]. This indicates that litter development depends on the quality of follicles, which, in turn, is defined during the period prior to insemination. However, it remains unknown if follicles are affected by different lengths of WII within the first follicular phase after weaning, or if the length of lactation affects PBW, CV_PBW_ and the occurrence of IUGR in the subsequent litter.

Following insemination of the sow, a favorable intrauterine environment is important for litter development. This involves the optimal nutritional status of the sow to ensure the growth and development of sufficient placentas. An insufficient placenta is obstructive for fetal growth and development [11], and the size and efficiency of the placenta can affect PBW, CV_PBW_ [12] and indications of IUGR [11]. Crowding in the uterus is a limiting factor for the development of both the placenta and fetus [13], and increasing litter size is related to a decrease in PBW [5,7,14,15,16,17], as well as to an increase in CV_PBW_ [5,7,17,18] and liveborn IUGR piglets [3,16]. The intrauterine environment also seems to be affected by the parity of the sow since PBW [6,7,17], CV_PBW_ [17,18,19] and the occurrence of liveborn IUGR piglets [16,20] differ between sows of different parities, although results are ambiguous.

Fetal development is highly affected by the nutritional status of the sow [21], and therefore the feeding method used during gestation could also affect it. Three commonly used feeding systems for gestating sows in Denmark are free-access stall feeding (Stall), floor feeding (Floor) and electronic sow feeding (ESF). Such systems present a lot of potential differences between them, including different levels of competition at feeding. This could entail that some sows do not get to eat their entire daily ration, and end up lacking nutrients needed for fetal growth. A compromised feed intake could affect sow body condition, since restricted feeding of gestating sows results in less backfat in late gestation [22]. However, studies of how the maternal body condition in late gestation affects PBW are not conclusive [19,23,24].

The objective of this paper was to identify possible risk factors affecting PBW, CV_PBW_ and the occurrence of IUGR in newborn piglets of hyperprolific Danish sows. Investigated factors included: (1) the duration of previous lactation, (2) the length of the interval from weaning to insemination, (3) the length of gestation, (4) litter size, (5) parity, (6) backfat thickness in late gestation and (7) the type of feeding system in the gestation unit.

## 2. Materials and Methods

The study was conducted on 12 commercial Danish pig herds with herd populations ranging from 800 to 3050 sows. Recordings were made successively over the three days with the most farrowings in one given week in each herd, during the period from mid-September to mid-December 2019. On average, 40–76% of all farrowings in the weekly farrowing batch at the herds were recorded.

### 2.1. Ethics Statement

All animals originated from commercial production facilities. No measurements were made that were outside of the standard industry animal husbandry techniques, and the animals were cared for in compliance with local legal standards. The health and welfare of all animals were monitored throughout the sampling days by farm staff, according to the farms’ standard operating protocols and veterinary recommendations. For more detailed health status of farms, see Bahnsen et al. [25].

### 2.2. Animals and Management

The study included 9652 piglets (8677 liveborn) from 452 litters. The sows were a crossbreed of Danish Landrace × Danish Yorkshire, artificially inseminated with semen from DanBred Duroc boars, and were between their 1st and 10th parity (mean ± SD; 3.84 ± 1.97). The herds were selected among those that used free-access feeding stall (Stall), floor feeding (Floor) or electronic sow feeding (ESF) in the gestation unit. Gilts in herd A were not housed in a stall system, but in pens with nine gilts/pen, and fed by liquid feed in a trough. The feed system, number of sows, average level of total born piglets per litter and number of litters and piglets included in the study are shown in Table 1 for the individual herds. Further information of the herds is presented in Supplementary Table S1.

**Figure 1:**
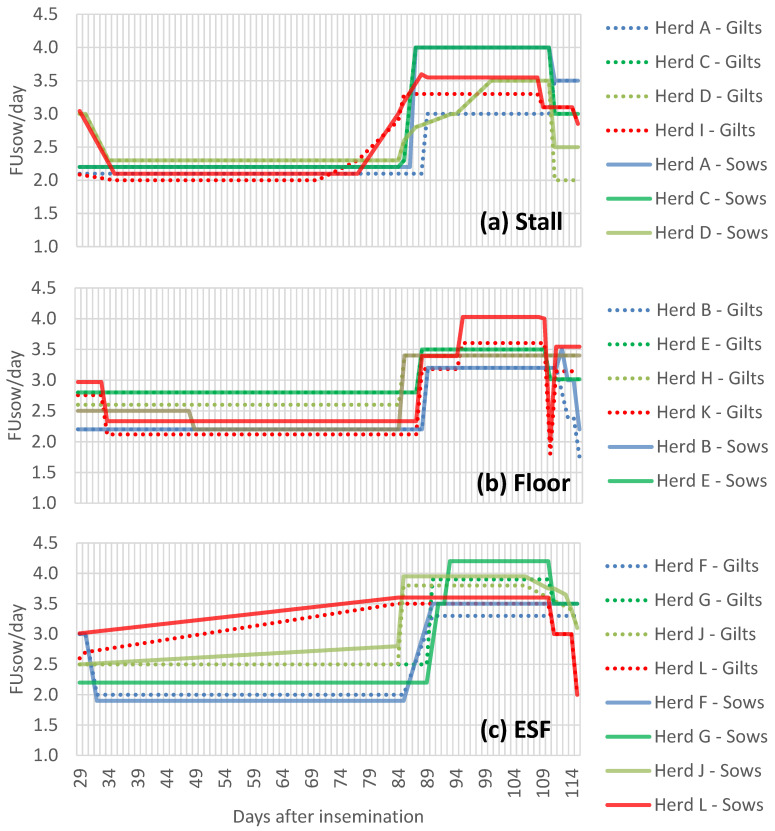
Feeding levels from day 29 to 115 of gestation for gilts and sows of normal body condition at the individual herds of the different feed systems (**a**–**c**). Stall describes free-access stalls, Floor describes floor feeding and ESF describes electronic sow feeding scheme.

Daily management routines were performed as usual on the individual herds, and recordings were collected early in the morning to prevent disturbing the work routines of the employees. In all herds, sows were housed in a crated system through the nursing period, and confined in stalls, either locked or free-access, for four weeks after insemination. Group size and number of daily feedings in the gestation unit can be found in Bahnsen et al. [25]. Gestation diets were formulated to meet or exceed the feed recommendations for gestating sows in all herds [26], and a reduced version of those formulations can also be found in Bahnsen et al. [25]. Feeding levels for gilts and sows from day 29 to 115 of gestation at each herd are illustrated in Figure 1. The feeding level presented for sows is the one used for sows of normal body condition at each herd. It was not possible to obtain information about which sows have been fed a higher or lower feed level in relation to their body condition. Therefore, some sows could have received a higher or lower feeding level during gestation than assumed.

### 2.3. Recordings

Recordings of piglets were carried out as soon as possible after farrowing had ended and before litter equalization. Piglets were no older than 24 h at the time of weighing and IUGR scoring. The definition of IUGR piglets and a picture of the categories have been published elsewhere (see [25] for details and the distinction between sIUGR, mIUGR and normal piglets according to shape of head and hind part). Briefly, piglets were scored as either normal, mild IUGR (mIUGR) or severe IUGR (sIUGR). The parameters for sIUGR and mIUGR are based on modified characteristics from Chevaux et al. [20], Hales et al. [4] and Engelsmann et al. [27]. The primary parameters characterizing IUGR piglets were defined steep/dolphin-like forehead, narrow hind part and low birth weight (below 1100 grams). Secondary parameters were defined as bulging eyes, wrinkles perpendicular to the mouth, spiky hair and unstable mobility. Piglets characterized as sIUGR piglets showed all primary parameters distinctively, had at least one of the secondary parameters and a weight of no more than 1050 g. As for mIUGR piglets, they had the primary parameters with a weight of maximum 1100 g, and no more than one of the secondary parameters. A normal piglet had none of the parameters and weighed more than 650 g.

The following was noted for each sow with a newborn litter: sow ID, date of farrowing, parity, backfat thickness, and number of total born, liveborn, stillborn and mummified piglets. When possible, both live- and stillborn piglets were individually scored as either normal, mIUGR or sIUGR, and the sex of those piglets was recorded. Some dead piglets were removed before registrations could be carried out, and in those cases, the number of stillborn piglets was only counted by employees, and sex and IUGR score were not noted. Dead piglets were classified as either stillborn or “liveborn but dead”, and if possible, the death cause was noted for “liveborn but dead” piglets. Dead and wet fully formed piglets with periople still present on the hooves were noted as stillborn. Test of inflation of the lung tissue was not performed. Piglets were noted as liveborn but dead if the above-mentioned criteria of stillborn piglets were not met. The reason of death was noted as either (1) crushed, if visible trauma or subcutaneous edema appeared on any part of the body; (2) euthanized, if clear signs of head trauma due to euthanization were visible; or (3) others, if no signs of either (1) or (2) could be detected.

All liveborn (including “liveborn but dead”) piglets were individually weighed by placing the piglet in a bucket hanging on a digital hanging scale (5 g weight interval) (Ryom Digital Hanging Scale, Hatting Agro, Horsens, Denmark). To minimize risk of disease spreading, a new but similar scale was used at each herd. The accuracy of each scale was tested, and a deviation of 25 g was found.

Backfat thickness was measured at the P2 site and by the same person at every herd. To minimize risk of disease spreading, the herds’ own backfat meter was used, which resulted in the use of different types of backfat meters. At herd A, C, E, G, H, I, J, K and L, the backfat meter model was “Lean-meater” (Lean Meter, Renco Corporation, MN, USA). At herd D it was an “Anyscan” (SONGKANG GLC Co., Ltd., Seongnam City, Korea) backfat meter and at herd F it was a “Sonograder II” (Renco Corporation, Golden Valley, MN, USA) backfat meter. In herd B, backfat was not measured because of missing backfat meter.

Additional information of the sows was collected from either direct data access of the management system using Cloudfarms (Bratislava, Slovakia) or via a webbackup, from PigVision (AgroVision, Hedensted, Denmark). This included the number of days from last weaning to insemination, length of previous lactation and length of gestation. Feed curves and diet formulations were collected at the herds.

### 2.4. Statistical Analysis

All statistical data analysis was performed in RStudio Version 1.2.503 © 2009–2019, and analysis of different models were tested by the ANOVA function. For linear mixed models, package nlme was used. For each mixed model, the random effect structure was assessed using Restricted Maximum Likelihood (REML) estimation. Results were presented as least square means except for the descriptive data, which are presented as the mean plus or minus the standard deviation of the mean, followed by the range (minimum and maximum). Statistical significance was accepted at *p* < 0.05, and 0.05 < *p* < 0.10 was considered as a tendency. All variables that showed a significant effect or a tendency were kept in the models.

#### 2.4.1. Analysis of Risk Factors for PBW, CV_PBW_ and IUGR

Analysis of PBW and CV_PBW_ was based on liveborn piglets only, since stillborn piglets were not weighed. PBW was analyzed for the individual piglet, whereas the parameters CV_PBW_, sIUGR and mIUGR were investigated as within-litter percentages. CV_PBW_ describes the coefficient of variation of PBW of liveborn piglets within the litter. Missing information of IUGR score caused 72 L to be excluded in this analysis. Furthermore, some piglets did not have any siblings categorized as sIUGR or mIUGR, which resulted in zero percentage of these measures in some litters. This caused the data to be zero-inflated and not normally distributed; therefore, the litters with 0% IUGR were excluded from the analysis of risk factors. Out of the 378 L with no missing values for IUGR score, 86 L had no sIUGR piglets and 69 L had no mIUGR piglets. Therefore, the risk analysis was based on 292 and 309 L for sIUGR and mIUGR, respectively.

Different models were built to analyze PBW, CV_PBW_ and IUGR for sows of 1st parity and 2nd to 9th parity, since the parameters length of lactation in last litter (LD) and WII were not applicable for sows of 1st parity. Furthermore, the parameter gestation days (GD) was not included in the analysis for sows of 1st parity because there were too few observations outside of the 1st and 3rd quartile (116, 118) to make a sensible difference for the outcomes. Parity 10 was also excluded, since these observations only consisted of one sow. Parity was divided into three groups: 1st, 2nd to 4th and ≥5th. Herd B was excluded from the analysis of the effect of backfat thickness, since backfat was not recorded in this herd. Analysis of the effect of WII was limited to sows with WII between 1 and 10 days, since the focus of the study was the first follicular phase, which ends within the first 10 days after weaning. GD was limited to 115–119 days for 2nd–9th parity sows because observations outside this interval were considered faulty recordings. Excluded data were included in the analysis again for re-testing, if the current variable was significant. This was performed to ascertain that our results did not change dramatically, thus arbitrarily obtaining significance through the removal of “undesirable” observations. The effect of gender was only included in the analysis for PBW, since the CV_PBW_ and IUGR were based on within-litter percentage. A correlation between CV_PBW_ and the average PBW was calculated by Pearson’s product-moment correlation.

##### Analysis for Sows of 1st Parity

The following model was used to estimate the effect of litter size, sex and backfat on PBW, sIUGR, mIUGR and CV_PBW_:

Y_*ijk*lm_ = *μ* + *α*_*i*_ + *β*_j_ + η_k_ + H_(l)_ + F_(m)_+ *ε*_*ijk*__lm_ (1)

where Y_*ijk*lm_ is the response variable (PBW, sIUGR, mIUGR, CV_PBW_), *α*_*i*_ is the fixed effect of litter size (*i* = [8, 9, …, 25]) as a covariate, *β_j_* is the fixed effect of sex of piglet (*j* = [male, female]), η_k_ is the fixed effect of backfat (*k* = [9, 10, …, 22]) explained as a covariate, H_l_ is the random effect of the herds (*l* = [A, B, …, L]), F_m_ is the random effect of the feed system (*q* = [Stall, Floor, ESF]), and *ε*_*ijk*__lm_ is the residual error component, which is assumed to be independent and normally distributed. Reduction of the model resulted in different final models for each response variable:

The final model used to investigate PBW:

Y_*ij*lm_ = *μ* + *α*_*i*_ + *β*_j_ + H_(l)_ + F_(m)_+ *ε*_*ij*__lm_

The final model used to investigate sIUGR and mIUGR:

Y_*ij*lm_ = *μ* + H_(l)_ + F_(m)_+ *ε*_*ij*__lm_

The final model used to investigate CV_PBW_:

Y_*ij*lm_ = *μ* + η_n_ + H_(l)_ + F_(m)_ + *ε*_*ij*__lm_

The following model was used to estimate differences in PBW, sIUGR, mIUGR and CV_PBW_ between the feed systems:

Y*_ijkl_* = *μ* + *α*_*i*_ + *β_j_* + η_k_ + *δ*_l_ + *ε*_*ijk*_*_l_*

The variables are the same as in Equation (1), apart from *δ_l_*, which is the fixed effect of the categorical variable feed system (*l* = [Stall, Floor, ESF]), and the herd effect is excluded because herds are nested within the feed systems. *ε*_*ijk*_*_l_* is the residual error component, which is assumed to be independent and normally distributed. The final models to analyze the differences between the feed systems were the same as the ones to test the effect of the other variables for each response variable, apart from the variable feed system being a fixed effect and that the herd effect was removed.

The following model was used to estimate the differences in PBW, sIUGR, mIUGR and CV_PBW_ between herds:

Y_*jkl*_ = *μ* + *α*_*i*_ + *β_j_* + η_n_ + κ_l_ + *ε*_*ijk*_*_l_*

The variables are the same as in Equation (1), apart from κ_l_, which is the fixed effect of the categorical variable herd (*l* = [A, B, …, L]), and the feed system effect is excluded because herds are nested within the feed systems. *ε*_*ijk*_*_l_* is the residual error component, which is assumed to be independent and normally distributed. The final models used to analyze the differences between herds were the same as the ones to test the effect of the other variable for each response variable, apart from the variable herd being a fixed effect and that the feed system effect was removed.

##### Analysis for Sows of 2nd to 9th Parity

The following model was used to estimate the effect of litter size, sex, GD, parity, backfat, WII and LD on PBW, sIUGR, mIUGR and CV_PBW_:

Y_*ijklmnopq*_ = *μ* + *α*_*i*_ + *β_j_* + *γ*_*k*_ + ζ_l_ + η_m_ + θ_n_ + ι_o_ + H_(p)_ + F_(q)_ + *ε*_*ijk*_*_lmnopq_* (2)

where Y_*ijklmnopq*_ is the response variable (PBW, sIUGR, mIUGR, CV_PBW_) and *μ* is the overall mean, *α*_*i*_ is the fixed effect of litter size (*i* = [4, 5, …, 34]) explained as covariate, *β_j_* is the fixed effect of the categorical variable “sex of piglet” (*j* = [male, female]), *γ_k_* is the fixed effect of GD (k = [115, 116, …, 119]) explained as covariate, ζ_l_ is the fixed effect of factor parity (l = [2–4; 5–9]), η_m_ is the fixed effect of backfat (m = [8, 9, …, 27]) explained as covariate, θ_n_ is the fixed effect of WII (n = [1, 2, …, 8]) explained as covariate, ι_o_ is the fixed effect of LD (o = [11, 12, …, 64]) explained as covariate, H_(p)_ is the random effect of the herd (p = [A, B, …, L]), F_(q)_ is the random effect of the feed system (q = [Stall, Floor, ESF]) and *ε_ijklmnopq_* is the residual error component, which is assumed to be independent and normally distributed. Reduction of the model resulted in different finals models for each response variable:

The final model used to investigate PBW:

Y_*ijlnopq*_ = *μ* + *α*_*i*_ + *β_j_* + ζ_l_ + θ_n_ + ι_o_ + H_(p)_ + F_(q)_ + *ε*_*ij*_*_lnopq_*

The final model used to investigate sIUGR:

Y*_inpq_* = *μ* + *α*_*i*_ + θ_n_ + H_(p)_ + F_(q)_ + *ε*_*i*_*_npq_*

The final model used to investigate mIUGR:

Y*_ipq_* = *μ* + *α*_*i*_ + H_(p)_ + F_(q)_ + *ε*_*i*_*_pq_*

The final model used to investigate CV_PBW_:

Y*_ilmpq_* = *μ* + *α*_*i*_ + ζ_l_ + η_m_ + H_(p)_ + F_(q)_ + *ε*_*i*_*_lmpq_*

The following model was used to estimate the differences in PBW, sIUGR, mIUGR and CV_PBW_ between the feed systems:

Y*_ijklmnop_* = *μ* + *α*_*i*_ + *β_j_* + *γ*_*k*_ + ζ_l_ + η_m_ + θ_n_ + ι_o_ + *δ*_p_ + *ε*_*ijk*_*_lmnop_*

where Y_*ijklmnop*_ is the response variable (PBW, sIUGR, mIUGR, CV_PBW_). The rest of the variables are the same as in the analysis of Equation (2), apart from δ*_p_*, which is the fixed effect of the categorical variable feed system (*p* = [Stall, Floor, ESF]), and herd is not included since herds are nested within the feed systems. *ε*_*ijk*__*lmnop*_ is the residual error component, which is assumed to be independent and normally distributed. The final models to analyze the differences between the feed systems were the same as the ones to test the effect of the other variables for each response variable, apart from the variable feed system being a fixed effect and that the herd effect was excluded.

The following model was used to estimate the differences in PBW, sIUGR, mIUGR and CV_PBW_ between herds:

Y*_ijklmnop_* = *μ* + *α*_*i*_ + *β_j_* + *γ*_*k*_ + ζ_l_ + η_m_ + θ_n_ + ι_o_ + κ_p_ + *ε*_*ijk*_*_lmnop_*

where Y_*ijklmnop*_ is the response variable (PBW, sIUGR, mIUGR, CV_PBW_). The rest of the variables are the same as in the analysis of Equation (2), apart from κ_p_, which is the fixed effect of the categorical variable herd (*p* = [A, B, …, L]), and feed system is not included since herds are nested within the feed systems. *ε_ijk__lmnop_* is the residual error component, which is assumed to be independent and normally distributed. The final models to analyze the differences between the herds were the same as the ones to test the effect of the other variables for each response variable, apart from the variable herd being a fixed effect and that the feed system was excluded.

## 3. Results

### 3.1. Descriptive Results

The averages and minimum and maximum values of the variables across the 12 herds were 3.8 (1 to 9) for parity, 21.3 total born piglets per litter (4 to 34) for litter size, 16.1 mm (8 to 27) for backfat thickness, 117.2 days (115 to 119) for GD, 4.1 days (1 to 8) for WII and 30.6 days (11 to 64) for LD.

Of the registered piglets, 87.5% were liveborn and alive at registration, and 2.4% were recorded as liveborn but dead at the time of data collection. Of the piglets included in the study, 10.5% were not weighed, and of these, 10.1% were stillborn. The remaining were liveborn but dead and were removed by the staff before weighing was carried out. Average PBW was 1235 ± 335 g, ranging from 290 g to 2910 g. In total, 11.0% of the piglets were defined as sIUGR, with an average weight of 699 ± 141 g ranging from 290 g to 1025 g, 11.5% were defined as mIUGR, with an average weight of 905 ± 94 g ranging from 535 g to 1100 g, and 75.5% were defined as normal, with an average weight of 1357 ± 265 g ranging from 610 g to 2910 g. The last 2% of the piglets were not identified. Of the stillborn piglets, 18.7% were defined as sIUGR, 14.8% as mIUGR and 66.5% as normal piglets.

The statistical analysis of the within-litter percentage of IUGR piglets showed the following: the prevalence of sIUGR was on average 10.9 ± 10.1% ranging from 0.0% to 52.9%, the prevalence of mIUGR was 11.8 ± 10.1% ranging from 0.0% to 47.8%, and 77.3 ± 16.6% ranging from 23.5% to 100% of the piglets in a litter were recorded as normal piglets. Out of the 378 L with no missing values for IUGR score, 32 L had only normal piglets.

**Figure 2:**
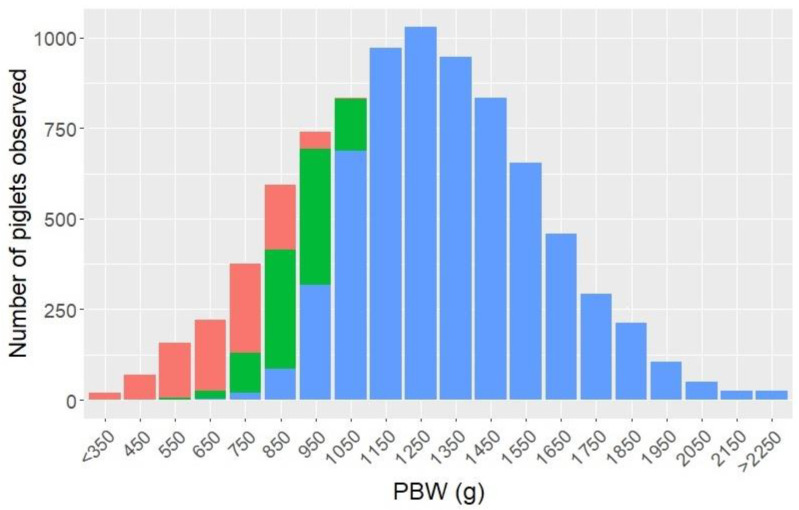
Number of liveborn piglets classified as normal (blue), mild IUGR (mIUGR (green)) and severe IUGR (sIUGR (red)), divided by birth weight intervals (PBW), where values on the *x*-axis cover ± 50 g.

Figure 2 illustrates the number of liveborn piglets of each IUGR score observed in relation to weight intervals. As the figure shows, the birth weights of the observed piglets followed a normal distribution, and both IUGR piglets and normal piglets were observed in the lower weight intervals, although a large proportion of those were classified as IUGR.

In all herds, at least one liveborn piglet had a PBW of less than 500 g, and one piglet had a PBW above 2100 g. Upper and lower limits for liveborn piglets in each herd are shown in Table 2. The average CV_PBW_ was 22.9 ± 5.5%, ranging from 7.1% to 38.3%.

**Figure 3:**
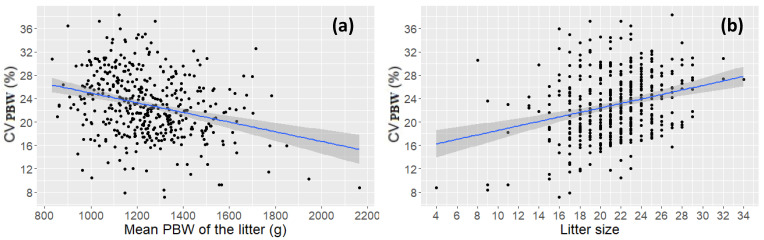
Relationship between (**a**) mean piglet birth weight (PBW) of the litter and within-litter percentage of PBW (CV_PBW_) across parities (n = 451). The fitted regression: CV_PBWi_% = 33.22 − 0.0083 * MeanPBW_i_+, *ε*~N(0, 5.131^2^). The correlation was −0.286 (*p* < 0.001), and (**b**) litter size and CV_PBW_ across parities (n = 451). The fitted regression: CV_PBWi_% = 17.34854 + 0.38800 * LS_i_+ *ε*_*i*_, *ε*~N(0, 5.14^2^). The correlation was 0.265 (*p* < 0.001).

A weak negative correlation was found between CV_PBW_ and the average PBW (−0.286; *p* < 0.001), and a positive correlation between CV_PBW_ and litter size (0.265 *p* < 0.001), which is shown in Figure 3.

### 3.2. Risk Factors

**Figure 4:**
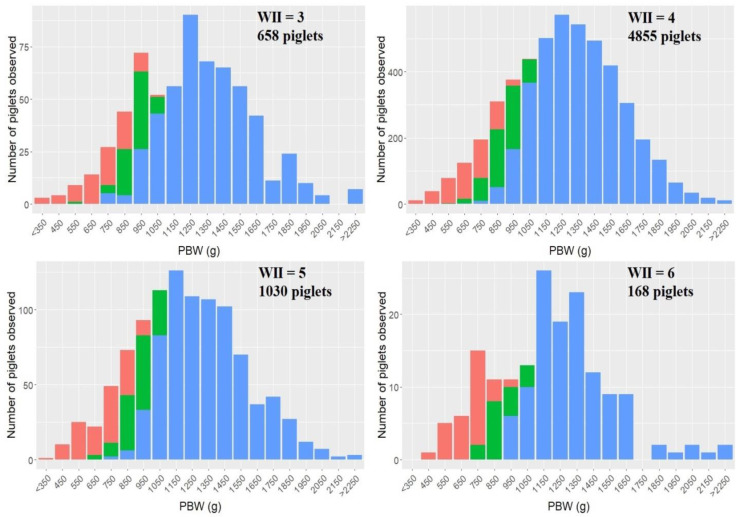
Distribution of piglets by piglet birth weight (PBW) classes of different weaning to insemination intervals (WII) with the number of piglets observed of each WII and classification of piglets as either normal (blue), mild IUGR (mIUGR (green)) or severe IUGR (sIUGR (red)). Values on the *x*-axis cover ±50 g. It should be noted that the majority of piglets were born of sows with a WII of four days, and the *y*-axis are differently proportioned.

The effects of WII, GD, LD and parity were only analyzed for sows of 2nd to 9th parity. PBW decreased by 25.8 g with increasing WII (*p* < 0.001), which is visible in Figure 4. However, WII had no effect on the occurrence of sIUGR piglets, mIUGR piglets or CV_PBW_.

There was a tendency of increasing LD to decrease PBW (*p* = 0.06) by 1.0 g for each additional day in the LD period. LD did not have a significant effect on either the occurrence of sIUGR, mIUGR nor CV_PBW_. GD had no effect on PBW, the occurrence of sIUGR, mIUGR nor CV_PBW_. Sows of 5th parity and older had 49.1 g lighter piglets (*p* < 0.001) and 1.39% higher CV_PBW_ (*p* < 0.05) than sows of 2nd to 4th parity. Parity had no significant effect on the occurrence of sIUGR or mIUGR piglets. Male piglets were significantly heavier than female piglets for both sows of 1st parity and sows of 2nd to 9th parity (50.5 g; *p* < 0.01 and 36.1 g; *p* < 0.001, respectively).

**Figure 5:**
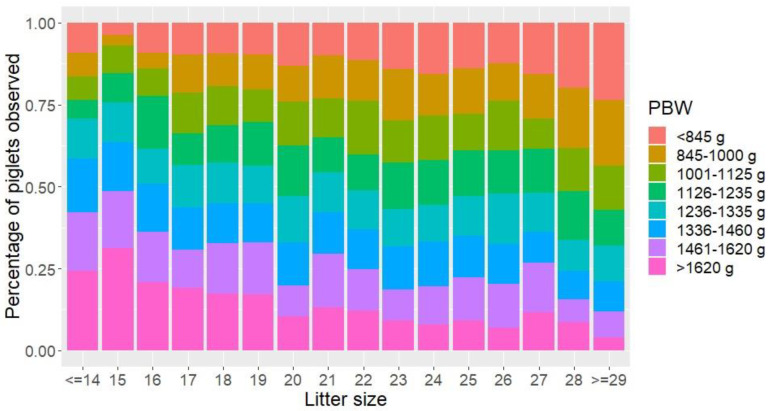
Percentage of liveborn piglets in different piglet birth weight (PBW) categories, showed by different colors, with increasing litter size across parities. The weight categories are based on quartiles, so there is an equal number of piglets in each PBW category.

Litter size had a significant effect on PBW for sows of 1st parity (*p* < 0.001) and sows of 2nd to 9th parity (*p* < 0.001). With each additional piglet in a litter, average PBW decreased by 19.5 g for sows of 1st parity and 21.7 g for sows of 2nd to 9th parity. From Figure 5, it appears that across parities the percentage of liveborn piglets in lower birth weight classes increases when the litter size increases.

**Figure 6:**
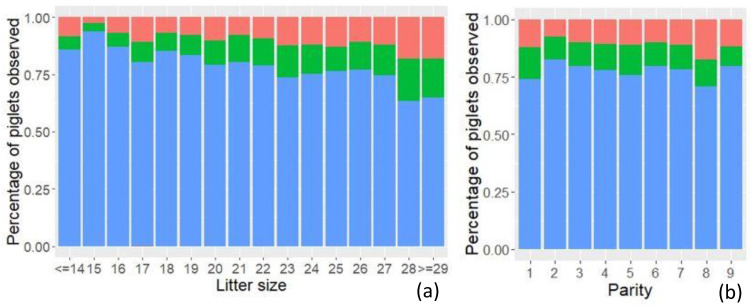
Percentage of normal (blue), mild IUGR (mIUGR (green)) and severe IUGR (sIUGR (red)) piglets with (**a**) increasing litter size and (**b**) of different parities.

For sows of 1st parity, litter size did not influence the occurrence of sIUGR or mIUGR piglets. For the sows of 2nd to 9th parity, an increased litter size resulted in an increased occurrence of sIUGR (*p* < 0.001) and mIUGR (*p* < 0.01), with 0.68% and 0.50% per further sibling, respectively. As illustrated in Figure 6, both mIUGR and sIUGR piglets were present in both small and larger litters, and in litters from sows of all parities. Litter size had no effect on CV_PBW_ for sows of 1st parity, whereas for sows of 2nd to 9th parity, one additional piglet in the litter increased CV_PBW_ with 0.38% (*p* < 0.001).

The backfat thickness at farrowing had no effect on PBW or on the prevalence of sIUGR or mIUGR piglets for sows of 1st parity, or 2nd to 9th parity. However, increasing backfat thickness increased CV_PBW_ with 0.49% per mm for 1st parity sows (*p* < 0.05), and 0.18% per mm for sows of 2nd to 9th parity (*p <* 0.05).

An overview of the effect of the tested risk factors, apart from the feed system, is presented in Table 3.

Feed system affected PBW and the percentage of sIUGR piglets, but not CV_PBW_ or the percentage of mIUGR piglets (Table 4). For 1st parity sows, PBW differed between Stall and Floor (*p* < 0.001), between Stall and ESF (*p* < 0.001) and between Floor and ESF (*p <* 0.01). Sows of 1st parity of Floor farrowed the heaviest piglets and sows of Stall the lightest piglets (Table 4). For sows of 2nd to 9th parity, sows of ESF had a significantly lower PBW than for sows of Stall (*p* < 0.001) and Floor (*p* < 0.001) (Table 4), but there was no difference between sows of Stall and Floor.

There was no difference in the occurrence of sIUGR or mIUGR between the feed systems for sows of 1st parity. For sows of 2nd to 9th parity, there was a significantly higher occurrence of sIUGR piglets in Stall compared to Floor (*p* < 0.05) and the ESF system (*p* < 0.05). There was no difference in the occurrence of sIUGR piglets between Floor and ESF. For the occurrence of mIUGR, there was no difference between feed systems for sows of 2nd to 9th parity.

**Figure 7:**
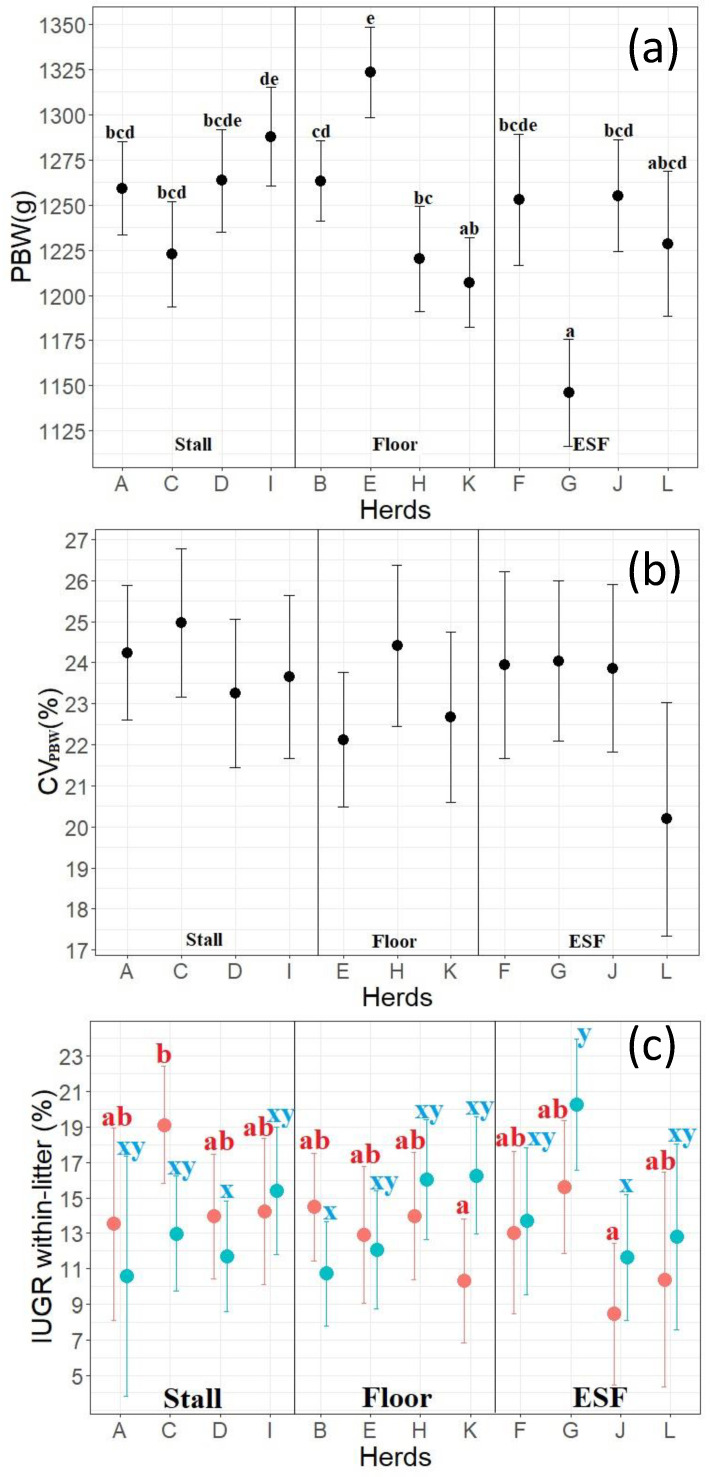
LSmeans of (**a**) piglet birth weight (PBW), (**b**) within-litter variation in PBW (CV_PBW_) and (**c**) within-litter proportion of either severe IUGR (sIUGR (red)) or mild IUGR (mIUGR (blue)) for 2nd to 9th parity sows of the different herds, described by the capital letters A–L on the *x*-axis, with visual separation by feed systems. Stall describes free-access feeding stalls, Floor describes floor feeding and ESF describes electronic sow feeding. ^a–e^ Values with different superscripts differ between herds for PBW and sIUGR (*p* < 0.05); ^x,y^ Values with different superscripts differ between herds for mIUGR (*p* < 0.05).

Within the feed systems, the level of PBW and occurrence of IUGR piglets of 2nd to 9th parity sows differed between herds (Figure 7). The CV_PBW_ of 2nd to 9th parity sows did not differ between either feed systems or herds (Table 3 and Figure 7).

## 4. Discussion

This study is based on data from 12 commercial Danish herds, and gives a broad view of PBW, CV_PBW_ and the occurrence of IUGR piglets in litters of hyperprolific sows with different housing and feeding systems. To our knowledge, this is the first study to compare the reproductive performance of sows of the investigated feed systems and report differences of not only PBW, but also the proportion of sIUGR piglets in litters.

The results show that a higher CV_PBW_ is connected to a lower PBW, where the averages for liveborn piglets in this study were 1235 g for PBW and 22.9% for CV_PBW_. This is in compliance with the literature, where CV_PBW_ is reported at 21% [17] and PBW at 1.2–1.3 kg/liveborn piglet for hyperprolific Danish sows [28]. The proportion of piglets defined as IUGR in this study (22.5%) is in line with earlier reports of Danish sows (20–32% [2,3]). A high variation in the within-litter proportion of IUGR piglets was observed, with some litters containing no IUGR piglets, as opposed to other litters with IUGR proportions as high as 76.5%.

The pre-insemination factor investigated, WII, decreased PBW by 25.8 g/piglet for each additional day, and to our knowledge, an effect of the length of WII within the first follicular phase after weaning on PBW has not been reported before. It should be noted that observations of sows with WII below four and above five are underrepresented as we only visited the herd on the largest farrowing days. It was expected that the length of WII might affect the quality of the follicles, and thereby the subsequent litter. However, WII did not affect CV_PBW_ or the occurrence of mIUGR or sIUGR piglets, which is difficult to explain without knowing the underlying mechanisms of the effect of the length of WII. The size of the follicles at weaning is determined during lactation, and sows with small follicles at weaning have a longer weaning to estrus interval [29]. The effect of WII seen in this study might reflect if the sow was in the right nutritional state during lactation and after weaning, when follicles developed. LH pulsatility is reported to be affected by IGF-1 [30] and plasma insulin [31,32,33]. However, other studies do not find plasma insulin to affect LH pulsatility [34]. Since plasma insulin concentrations vary with feeding and time of day, when compared to IGF-1, which is relatively stable [35], it is suggested that IGF-1 through feeding has a higher impact on LH pulsatility, and thereby follicle development, than insulin. Since the nutritional state of the sows was not studied directly in this study, further investigation is needed to clarify how the length of WII affects the subsequent litter.

Litter size was identified as a major predictor for PBW, CV_PBW_ and the occurrence of IUGR piglets. Increasing litter size caused a higher proportion of small piglets to be born at the cost of fewer heavy piglets, which is supported by the literature [5,7,17,18,36]. One additional piglet in the litter decreased PBW by around 20 g for sows of all parities, which is less than the 30–40 g per piglet reported in the literature [7,15,18]. CV_PBW_ increased with litter size, as reported in the literature [5,7,17,18]. However, litter size only affected CV_PBW_ of 2nd to 9th parity sows, and only with an increase of 0.38% per additional piglet in the litter, which is lower than earlier reported values of approximately 0.8% [7,18]. The differences in the magnitude of the effects of increasing litter size might be due to a higher prolificacy of the sows investigated in the current study. Litter size was the only risk factor identified to significantly affect the proportion of IUGR piglets of 2nd to 9th parity sows, whereas no risk factors were identified for 1st parity sows. The negative effects of high litter sizes could be connected to a lower uterine blood flow per fetus when litter size increases [37]. Intrauterine crowding and insufficient placentas could also be an effect of increased litter size since intrauterine crowding can result in a compromised placental development and thereby reduced fetal growth [13]. However, a high ovulation rate and more viable conceptuses do not result in lighter fetuses at day 25 to 44 of gestation [38] or growth retardation at day 27 [39]. The results of the effect of litter size on PBW, CV_PBW_ and IUGR of this study suggest that intrauterine crowding becomes a limiting factor for fetal development at a later stage of gestation.

Besides litter size and parity, backfat thickness at farrowing was found to affect CV_PBW_. The increasing effect on CV_PBW_ with higher backfat thickness at farrowing for all parities is, however, difficult to explain. Earlier studies found no effect of backfat at any point of gestation on CV_PBW_, but an increased PBW with increasing backfat thickness at late gestation (day 80 and 110) [19]. Other studies have reported a quadratic effect of backfat, with an optimum value of 19–20 mm in late gestation (day 109) to increase PBW, and of 17–20 mm to decrease the proportion of low birth weight piglets [24]. However, the backfat thickness at farrowing did not affect PBW or the occurrence of IUGR piglets in the present study. Backfat thickness at insemination affects backfat changes through gestation, where thin gilts (~12 mm) will stay different from fat gilts (~19 mm) and have lighter piglets [22]. Moreover, changes in backfat thickness through gestation are related to feed level, and gilts fed a high feed level farrow heavier piglets than gilts fed a restricted feed level [22]. Backfat at farrowing or in late gestation only indicates the body condition of the sow at that particular time and cannot reveal connections to the feed level of the sow or the backfat changes through gestation, which might have affected fetal development. Further investigation of backfat changes through gestation and how they relate to feed level could clarify the results of CV_PBW_ of the current study.

As for the effect of feeding systems, it was unexpected that gilts of Stall had the lowest PBW, since the gilts can escape fighting and feed undisturbed in that system [40]. The feed intake of young sows in ESF was expected to be limited by the fact that younger sows are not always able to consume their entire daily ration in one feeding and do not return for additional feedings [41,42]. However, earlier findings also reported that 1st parity sows of Stall farrow lighter piglets than 1st parity sows of ESF [42]. The study by Van der Peet-Schwering et al. [42] reported the same relationship for 2nd parity sows, but found no difference in PBW of 3rd parity sows in the two systems and did not investigate older sows. The comparison of the results of the current study is compromised since 2nd to 9th parity sows were investigated as a group. However, 2nd to 9th parity sows of ESF farrowed the lightest piglets compared to Stall and Floor in the current study, so the results seem to conflict with the study of Van der Peet-Schwering et al. [42]. The underlying circumstances for these results are unknown, but other management factors such as the actual feed consumption and utilization might explain the differences detected between feed systems for both sows of 1st and 2nd to 9th parity. The variations in PBW and the occurrence of IUGR piglets of sows of 2nd to 9th parity between herds within the feed systems also suggest that further investigation of other factors is needed to explain the observed differences. In addition, feeding systems will result in changes in stress, which has been linked to fetal development, and this was investigated more thoroughly in Bahnsen et al. [25] using the same piglet data but measuring cortisol levels during gestation on herd level. Here, sows from Stall had the lowest levels of cortisol compared to Floor and ESF, but with no differences in the percentage of IUGR piglets or PBW between the systems. Interestingly, one herd (herd G, ESF with deep straw) differed in both cortisol levels (was lower than the other herds) in Bahnsen et al. [25], but also surprisingly had lower PBW in the current study than the other ESF herds. This suggests that there are interactions and management factors that need to be further investigated to understand the underlying mechanisms at play.

## 5. Conclusions

The current study identified several risk factors for undesirable PBW, CV_PBW_ and the occurrence of IUGR piglets for hyperprolific sows with an average litter size of 21.3 piglets per litter. The main finding was that an increased litter size and increasing WII decreased PBW. Additionally, parity had an influence on PBW, CV_PBW_ and the occurrence of IUGR piglets with increased risks in older sows. The differences between feed systems are likely to be caused by underlying mechanisms that were not studied, since the level of PBW and the occurrence of IUGR varied between the individual herds within feed systems. Further investigation of the identified risk factors is needed to clarify underlying mechanisms.

## Figures and Tables

**Table 1:** Feed system, number of sows, average level of total born per litter and number of litters and piglets in the study of the individual herds (presented by letters A–L). Stall describes free-access feeding stalls, Floor describes floor feeding and ESF describes electronic sow feeding system.

	Stall				Floor				ESF			
Herd	A	C	D	I	B	E	H	K	F	G	J	L
Sows ^1^	1900	1250	1050	1150	3050	2500	1250	2000	800	1200	1400	1700
Total born/litter ^2^	21.2	20.8	21.2	19.9	20.1	18.8	21.1	19.9	19.5	19.5	19.4	20.0
Litters included in study	40	38	37	34	56	45	36	45	30	33	25	33
Total piglets included in study	896	769	791	741	1201	907	801	991	624	692	587	652
Liveborn piglets weighed in study	815	704	702	678	1053	853	713	895	549	611	485	619

^1^ Rounded to closest 50; ^2^ Information from production reports (1 January 2019 to 1 September 2019) of the herds.

**Table 2:** Average, minimum and maximum of piglet birth weight at each herd. Stall describes free-access feeding stalls, Floor describes floor feeding and ESF describes electronic sow feeding.

	Stall				Floor				ESF			
Herd	A	C	D	I	B	E	H	K	F	G	J	L
Mean (g)	1228	1217	1248	1265	1261	1339	1195	1210	1216	1162	1212	1217
Min. (g)	350	405	355	390	400	490	370	300	290	335	390	425
Max. (g)	2150	2445	2445	2215	2910	2515	2425	2180	2540	2335	2525	2240

**Table 3:** Overview of the effects of tested risk factors apart from feed systems ^1^.

		PBW (g)		sIUGR (%)		mIUGR (%)		CV_PBW_ (%)	
**First Parity Sows**	Litter size, increasing	↓	–19.5	NS		NS		NS	
	Backfat, increasing	NS		NS		NS		↑	0.49
	Male vs. female piglet	↑	50.5						
**Second to Ninth Parity Sows**	Litter size, increasing	↓	–21.7	↑	0.68	↑	0.50	↑	0.38
	Backfat, increasing	NS		NS		NS		↑	0.18
	Male vs. female piglet	↑	36.1						
	GD, increasing	NS		NS		NS		NS	
	WII, increasing	↓	–25.8	NS		NS		NS	
	LD, increasing	*↓	–1	NS		NS		NS	
	Parity ≥ 5	↓	–49.1	NS		NS		↑	1.39

^1^ The abbreviations are as follows: length of gestation in days (GD), weaning-to-insemination interval (WII) in days, length of lactation in days (LD), piglet birth weight (PBW), within-litter percentage of PBW (CV_PBW_), within-litter percentage of severe IUGR (sIUGR) and mild IUGR (mIUGR). *↓ represents decreasing tending effect (*p* = 0.05–0.10), ↑ represents increasing effect (*p* < 0.05) and ↓ represents decreasing effect (*p* < 0.05).

**Table 4:** LSmeans of piglet birth weight (PBW), within-litter proportion of severe IUGR (sIUGR) and mild IUGR (mIUGR) piglets and within-litter variation in PBW (CV_PBW_) of the different feed systems for sows of 1st and 2nd to 9th parity. Stall describes free-access feeding stalls, Floor describes floor feeding and ESF describes electronic sow feeding.

		PBW (g)		sIUGR (%)		mIUGR (%)		CV_PBW_ (%)	
	Feed System	Mean	SE	Mean	SE	Mean	SE	Mean	SE
**First Parity Sows**	Stall	1072 ^a^	15.5	14.7	2.5	17.9	2.8	22.1	1.30
	Floor	1205 ^c^	13.8	15.4	2.6	14.7	2.6	18.9	1.35
	ESF	1149 ^b^	11.9	16.2	2.2	18.7	2.4	20.0	1.09
**Second to Ninth Parity sows**	Stall	1259 ^y^	22.5	15.7 ^y^	1.0	13.0	0.9	24.1	0.5
	Floor	1257 ^y^	22.4	13.1 ^x^	0.9	13.5	0.8	22.9	0.6
	ESF	1216 ^x^	23.2	12.2 ^x^	1.1	14.8	1.0	23.3	0.6

^a,b,c^ Values within a column with different superscripts differ between feed systems for sows of 1st parity (*p* < 0.05); ^x,y^ Values within a column with different superscripts differ between feed systems for sows of 2nd to 9th parity (*p* < 0.05).

## Data Availability

Not applicable.

## Supplementary Materials

The following are available online at https://www.mdpi.com/article/10.3390/ani11092731/s1, Table S1: Herd information from production reports (1 January–1 September 2019) for the twelve farms included in the study (A–L). Stall describes free-access feeding stalls, Floor describes floor feeding and ESF describes electronic sow feeding.
