# Supplementary material for: Identifying Risk Factors for Low Piglet Birth Weight, High Within-Litter Variation and Occurrence of Intrauterine Growth-Restricted Piglets in Hyperprolific Sows
Source: Animals (Basel). 2021 Sep 18;11(9):2731. doi: 10.3390/ani11092731 (PMC8468730; doi:10.3390/ani11092731)
Supplement: Supplementary file 1 [file animals-11-02731-s001.zip › animals-1355360-supplementary.pdf]

animals

MDPI

Article

Identifying Risk Factors for Low Piglet Birth Weight, High Within-Litter Variation and Occurrence of Intrauterine Growth-Restricted Piglets in Hyperprolific Sows

Kristina V. Riddersholm [1,†], Ida Bahnsen [1,†], Thomas S. Bruun [2], Leonardo V. de Knegt [1] and Charlotte Amdi [1,*],

[1] Department of Veterinary and Animal Sciences, Faculty of Health and Medical Sciences, University of Copenhagen, 1870 Frederiksberg, Denmark; riddersholm95@hotmail.com (K.V.R.); idabahnsen@yahoo.dk (I.B.); lvdk@sund.ku.dk (L.V.d.K.)
[2] SEGES Danish Pig Research Centre, 1609 Copenhagen, Denmark; thsb@seges.dk
* Correspondence: ca@sund.ku.dk
† Joint first authors.

Table S1. Herd information from production reports (1st of January-1st of September 2019) for the twelve farms included in the study (A-L). Stall describes free-access feeding stalls, Floor describes floor feeding and ESF describes electronic sow feeding.

	Stall				Floor				ESF			
**Herd**	**A**	**C**	**D**	**I**	**B**	**E**	**H**	**K**	**F**	**G**	**J**	**L**
Year sows [1]	1900	1250	1050	1150	3050	2500	1250	2000	800	1200	1400	1700
Liveborn piglets/litter	19.2	18.9	18.6	17.9	18.2	17.3	18.9	18.2	18.4	17.7	17.1	18.5
Stillborn piglets/litter	2.0	1.9	2.6	2.0	1.9	1.5	2.2	1.7	1.1	1.8	2.3	1.5
Litters/year sow	2.23	2.36	2.37	2.36	2.36	2.29	2.34	2.34	2.34	2.23	2.24	2.36
Weaned piglets/year sow	35.5	38.6	36.3	35.4	37.8	35.8	36.0	35.5	37.6	31.6	34.2	37.8
Weaned piglets/weaning	12.1	13.0	13.4	12.7	15.2	12.5	11.1	11.8	13.6	11.3	11.8	13.3
Weaned piglets/litter	15.9	16.4	15.3	15.0	16.0	15.6	15.4	15.1	16.0	14.2	15.2	16.0
Piglets mortality before weaning (%)	17.2	13.2	18.0	14.6	11.1	10.0	18.9	16.9	12.5	19.9	11.2	12.9
Length of lactation (days)	36	30	29	28	24	28	29	30	29	29	31	28
Farrowing rate (%)	92.6	96.1	92.8	91.7	90.1	92.2	92.9	91.8	87.4	82.9	89.2	92.6
Return rate (%)	1.6	1.0	4.7	2.8	3.0	3.3	1.8	2.7	3.5	6.3	4.9	2.2
Weaning-to-insemination interval (days)	6	5	5	5	5	6	5	6	8	5	7	6
Home production (HP) or buying (B) young females	B	B	B	HP [2]	B	B	B	HP [3]	B	B	B	B

[1] Rounded to closest 50. [2] Zigzag-cross with nucleus management. [3] Own production by bought YY-sows.
